# Burden of psychiatric and somatic comorbidities in individuals with suicidal behavior: a nationwide Danish registry-based, observational study

**DOI:** 10.1192/j.eurpsy.2024.1781

**Published:** 2025-01-21

**Authors:** Mette Reilev, Jens-Jakob Kjer Møller, Elsebeth Stenager, Erik Christiansen

**Affiliations:** 1 Centre for Suicide Research, Odense, Denmark; 2 Clinical Pharmacology, Pharmacy, and Environmental Medicine, Department of Public Health, University of Southern Denmark, Odense, Denmark; 3 The Research Unit in Psychiatry – Child and Adults, Psychiatry in the Region of Southern Denmark, Aabenraa, Denmark; 4 OPEN – Open Patient Data Explorative Network, Odense University Hospital, Odense, Denmark

**Keywords:** comorbidity burden, epidemiology, suicide research

## Abstract

**Background:**

Many psychiatric and somatic comorbidities increase the risk of suicidal behavior, but the effect of co-existing comorbidities is sparsely elucidated. We described co-existence of psychiatric and somatic comorbidities and the influence of the combined comorbidity burden on the risk of suicidal behavior.

**Methods:**

We defined two case populations above 10 years in the Danish health registries: those who 1) died by suicide (2010–2020) and 2) had an incident suicide attempt (2010–2021). Co-existing somatic and psychiatric comorbidities and relative odds of suicidal behavior at increasing comorbidity burden were assessed.

**Results:**

Among 5.9 million Danish citizens (2021), 6,257 individuals died by suicide whereas 30,570 had an incident suicide attempt. More than half had ≥2 co-existing psychiatric and/or somatic comorbidities. Of those who died by suicide, 18% had co-existing mood disorders and stress disorders, while 5% had both mood disorders and cancer. An 88-fold increase of odds for attempting suicide and a 35-fold increase of odds for suicide were observed among those with the highest combined burden of somatic and psychiatric comorbidities relative to those without. The presence of somatic comorbidities seemed to protect against suicide in older individuals.

**Conclusions:**

Psychiatric and somatic comorbidities commonly co-exist in individuals with suicidal behavior. Higher combined burden of psychiatric and somatic comorbidities increased the odds of suicidal behavior, though the presence of somatic diseases had a potential protective effect on the risk of suicide in older individuals. This warrants collaboration and enhanced awareness of suicidal behavior risks across somatic and psychiatric departments.

## Introduction

The identification of individuals at high risk of suicidal behavior stands as a paramount imperative in public health and clinical practice to prevent suicide [1]. Despite extensive research efforts, it is difficult to distinguish those who act on suicidal thoughts from those who do not and simple suicide risk assessment models including single risk factors are deemed inadequate. This is partly due to the complex nature of suicide with intricate dynamic interactions between social, psychological, and biological determinants [2], and partly due to the low base rates of suicidal behavior. An extensive meta-analysis including the last 50 years of research in suicidal behavior evaluated the value of risk assessments and found that no single risk factors seem to hold a substantial clinical value if evaluated separately. Instead, a nuanced exploration of the interplay between several risk factors is needed to augment the clinical value [3].

A range of isolated psychiatric disorders and somatic diseases are known to increase the risk of suicidal behavior. To this end, individuals with psychiatric disorders like psychotic disorders and mood disorders have a many-fold higher risk of suicide (risk ratios 12–13), but also for some somatic diseases like epilepsy and concussion, the risk of suicide is correspondingly increased 2–3-fold [4]. Despite this, there is a paucity of studies evaluating whether co-existence of psychiatric and somatic comorbidities increases the risk of suicide to a magnitude relevant for risk assessment in clinical practice.

To adopt the most comprehensive and informative approach for risk assessment, detailed knowledge about co-existing somatic and psychiatric comorbidities in individuals with suicidal behavior is mandatory. In this study, we, therefore, aimed to map prevalence and co-existence of psychiatric disorders and somatic diseases in individuals with suicidal behavior and to determine whether the combined burden of somatic and psychiatric comorbidities affects the risk of suicide and suicide attempt.

## Methods

In this nationwide, registry-based, descriptive study, we described co-existence and burden of psychiatric disorders and somatic diseases among all Danish individuals above 10 years of age with suicidal behavior from 2010 to 2021.

### Data sources

The entire Danish population has access to tax-funded healthcare services regardless of age, sex, and income. This includes free access to both primary and secondary care. The National Health Service System stores individual-level information on provided healthcare services in nationwide health registries.

Through the unique personal identifier provided to all Danish citizens [5], data from the nationwide health registries were linked to individual-level data on income, civil status, and so forth from Statistics Denmark. The following four health registries were used: the Danish National Patient Registry, the National Prescription Registry, the Danish Civil Person Registry, and the Causes of Death Registry. The Danish National Patient Registry covers data on, for example, date of admission and the discharge diagnoses coded by the International Classification of Disease 10th version (ICD-10) [6] for all hospital admissions since 1977 and contacts to outpatient clinics and psychiatric wards since 1995. The National Prescription Registry records data on all redeemed prescription drugs by Danish citizens at outpatient pharmacies since 1995 and onward [7]. Among others, prescription data include the date of dispensing and the substance. Drugs are categorized according to the Anatomical Therapeutic Chemical (ATC) code, developed by the WHO for purposes of drug use statistics [8]. The Danish Civil Person Registry covers every Danish citizen and records data on vital status (date of birth and death) and migrations to and from Denmark [5]. The Danish Register of Causes of Death contains information on the date, cause, and mode of death (natural cause, suicide, homicide, or accident) [9].

### Study population

Case- and reference groups were built separately for each outcome. The case groups included all Danish citizens who had an incident suicide attempt from January 1, 2010, to December 31, 2021, and suicide from January 1, 2010, to December 31, 2020. Due to shorter lag time in the Danish National than in the Cause of Death Registry Patient Registry, more recent data were included in the analysis of suicide attempts. To inform the reader of prevalences and co-existences of comorbidities in similar groups of individuals in the background population, we established reference groups including individuals who had not (yet) experienced the outcome by matching in a ratio of 1:10 on age, sex, at the index dates of the corresponding cases. Individuals in the reference groups were assigned an index date identical to the outcome date of the corresponding case. To be included, individuals in the case- and reference groups were at least 10 years old at the outcome-/index date. Individuals were excluded if data source coverage was incomplete during a 10-year window prior to the outcome-/index date.

### Definition of suicide and suicide attempt

Two outcomes were considered: 1) suicide and 2) incident suicide attempt. Incident suicide attempt was defined as the first recording of diagnosis codes indicating deliberate self-harm using a 10-year look back. Acknowledging that some misclassification of suicide attempts may occur [10], a broader definition of suicide attempts was applied in a sensitivity analysis. In addition to deliberate self-harm, this definition also covered events of undetermined intent, accidental poisonings, and injuries to the lower forearm in individuals diagnosed with psychiatric disorders. A detailed description of coding algorithms used to define outcomes can be found in Supplementary Table S1.

### Definition of psychiatric and somatic comorbidities

The most common and/or severe chronic somatic diseases and psychiatric disorders were selected *a priori* by the author group. For a complete list hereof including corresponding coding algorithms, see Supplementary Table S2. A 10-year look back in the health registries was used to establish the existence of a disease. Only these prespecified diseases were included in the analyses described below. Throughout the paper, “co-existing comorbidities” refers to the recording of two or more somatic diseases and/or psychiatric disorders in the Danish National Patient Registry during this 10-year look back period.

### Statistical analysis

Individuals were characterized on the outcome-/index date in terms of sex, age, socio-economic factors, and history of somatic diseases and psychiatric disorders and hospital admissions.

Next, the 10 most common psychiatric disorders and somatic diseases and co-existence hereof were illustrated in a network graph, where the size of the nodes is proportional to the number of individuals with a given disease and the thickness of the links between the nodes to the number of individuals sharing both diseases. To avoid over-cluttering of the network graph, only diseases co-existing in more than 1% of individuals were visually linked to each other.

Finally, the crude odds ratios (ORs) of suicidal behavior given different burdens of psychiatric and somatic comorbidities on the outcome-/index date were visualized in a heat map with the count of somatic diseases (0, 1, 2, 3+) on the x-axis and the count of psychiatric disorders (0, 1, 2, 3+) on the y-axis. For each cell in the heat map, the odds for experiencing the outcome when having a specific combination of counts of psychiatric and somatic comorbidities was determined and the odds for experiencing the outcome when having zero somatic diseases and zero psychiatric diseases was used as reference, thus providing crude ORs. Crude ORs were calculated using conditional logistic regression, and can in this case be interpreted as risk ratios, since the outcome is rare and controls are drawn from the background population [11]. Each disease contributed in an unweighted manner, that is, ignoring that different diseases add differently to the risk of suicidal behavior. *Post hoc*, it was estimated whether the existence of somatic diseases (yes/no) modified the relative odds of experiencing the outcome if having 0, 1, or 2+ psychiatric diseases by including somatic diseases, psychiatric disorders, and the combination hereof as an interaction term.

All analyses were performed for the overall case- and reference groups built for each outcome. Analyses were repeated stratified by sex. For suicide attempts, a sensitivity analysis was conducted based on the broader definition hereof (see *Outcomes*).

### Other

According to Danish law, studies based entirely on registry data do not require approval from an ethics review board. [12] The study was registered at the repository of the University of Southern Denmark (notification number 11.773) and data were available from Statistics Denmark (project number 708951). Due to legal reasons, individual-level data cannot be shared by the authors.

## Results

In a population of 5.8 million Danish citizens (2020), 6,257 individuals died by suicide from 2010 to 2020 whereas 30,570 had a first suicide attempt from 2010 to 2021. Those who died by suicide were more often men (73% vs. 40% among individuals who attempted suicide) and older (median age 55 years vs. 31 years for individuals who attempted suicide; Table 1).

**Table 1.** Baseline characteristics

	Suicide case group	Suicide reference group	Suicide attempt case group^a^	Suicide attempt reference group
	6,257	62,570	30,570	305,699
*Demographics*				
Men	4,559 (72.9)	45,590 (72.9)	12,208 (39.9)	122,079 (39.9)
Women	1,698 (27.1)	16,980 (27.1)	18,362 (60.1)	183,620 (60.1)
Age [IQR]	55 [43–69]	55 [43–69]	31 [19–50]	31 [19–50]
70+ years	1,480 (23.7)	14,744 (23.6)	2,235 (7.3)	22,406 (7.3)
55–69 years	1,745 (27.9)	17,567 (28.1)	3,550 (11.6)	35,444 (11.6)
40–54 years	1,854 (29.6)	18,447 (29.5)	6,323 (20.7)	63,272 (20.7)
23–39 years	897 (14.3)	9,005 (14.4)	7,020 (23.0)	70,262 (23.0)
10–22 years	281 (4.5)	2,807 (4.5)	11,442 (37.4)	114,312 (37.4)
*Socioeconomics*				
Income				
<1. quartile	1,928 (30.8)	12,062 (19.3)	9,642 (31.5)	65,468 (21.4)
1.–2. quartile	1,584 (25.3)	12,138 (19.4)	7,080 (23.2)	55,785 (18.2)
2.–3. quartile	1,144 (18.3)	12,606 (20.1)	5,400 (17.7)	59,041 (19.3)
3.–4. quartile	845 (13.5)	12,907 (20.6)	4,597 (15.0)	64,047 (21.0)
>4. quartile	756 (12.1)	12,856 (20.5)	3,847 (12.6)	61,358 (20.1)
Married	1,989 (31.8)	33,697 (53.9)	5,778 (18.9)	92,750 (30.3)
Unmarried	4,268 (68.2)	28,873 (46.1)	24,792 (81.1)	212,949 (69.7)
*Educational level*				
Primary school	2,286 (36.5)	17,757 (28.4)	18,081 (59.2)	130,548 (42.7)
High school or vocational	2,536 (40.5)	26,953 (43.1)	8,456 (27.7)	105,834 (34.6)
Further education or bachelor	903 (14.4)	11,433 (18.3)	2,344 (7.7)	44,328 (14.5)
Master or PhD	353 (5.6)	4,964 (7.9)	551 (1.8)	17,596 (5.8)
Unknown	178 (2.8)	1,403 (2.2)	1,120 (3.7)	7,209 (2.4)
*Occupation*				
Student	264 (4.2)	3,115 (5.0)	9,473 (31.0)	108,817 (35.6)
Retired	2,955 (47.2)	22,275 (35.6)	6,838 (22.4)	39,837 (13.0)
Working	2,020 (32.3)	33,068 (52.8)	7,577 (24.8)	134,898 (44.1)
Unknown	1,018 (16.3)	4,112 (6.6)	6,681 (21.9)	22,147 (7.2)
*History of psychiatric disease*				
Hospitalization at psychiatric department during previous year^b^	1,377 (22.0)	311 (0.5)	6,188 (20.2)	1,204 (0.4)
Hospitalization at psychiatric department within previous 30 days^b^	484 (7.7)	42 (0.1)	2,354 (7.7)	149 (0.0)
Mood disorders	3,095 (49.5)	6,617 (10.6)	14,797 (48.4)	24,949 (8.2)
Anxiety	469 (7.5)	723 (1.2)	3,022 (9.9)	4,826 (1.6)
PTSD	105 (1.7)	231 (0.4)	720 (2.4)	912 (0.3)
Other stress disorders than PTSD	1,453 (23.2)	1,363 (2.2)	9,021 (29.5)	8,578 (2.8)
Obsessive-compulsive disorder	57 (0.9)	110 (0.2)	621 (2.0)	1,458 (0.5)
Psychotic disorders	736 (11.8)	700 (1.1)	3,342 (10.9)	2,974 (1.0)
ADHD	174 (2.8)	338 (0.5)	2,215 (7.2)	3,949 (1.3)
Eating disorder	66 (1.1)	79 (0.1)	1,109 (3.6)	1,860 (0.6)
Substance abuse	674 (10.8)	587 (0.9)	3,287 (10.8)	2,389 (0.8)
Alcohol abuse	1,351 (21.6)	2,096 (3.3)	6,356 (20.8)	6,734 (2.2)
Dementia	130 (2.1)	998 (1.6)	309 (1.0)	1,570 (0.5)
Borderline personality disorder	204 (3.3)	114 (0.2)	1,655 (5.4)	1,057 (0.3)
Other personality disorders than borderline	416 (6.6)	363 (0.6)	2,487 (8.1)	2,211 (0.7)
Autism	76 (1.2)	135 (0.2)	1,052 (3.4)	2,402 (0.8)
*History of somatic diseases*				
Hospitalization at somatic department during previous year^b^	2,150 (34.4)	6,687 (10.7)	10,383 (34.0)	22,373 (7.3)
Hospitalization at somatic department within previous 30 days^b^	889 (14.2)	823 (1.3)	4,976 (16.3)	2,554 (0.8)
Chronic lung disease	529 (8.5)	2,967 (4.7)	2,344 (7.7)	11,426 (3.7)
Ischemic heart disease	562 (9.0)	4,867 (7.8)	1,462 (4.8)	8,353 (2.7)
Heart failure	240 (3.8)	1,628 (2.6)	445 (1.5)	2,314 (0.8)
Stroke	426 (6.8)	2,423 (3.9)	1,053 (3.4)	4,240 (1.4)
Cancer	644 (10.3)	4,099 (6.6)	1,253 (4.1)	8,788 (2.9)
Inflammatory bowel disease	102 (1.6)	704 (1.1)	398 (1.3)	2,938 (1.0)
Rheumatoid arthritis	62 (1.0)	594 (0.9)	210 (0.7)	1,639 (0.5)
Hospital-diagnosed kidney disease	153 (2.4)	1,005 (1.6)	372 (1.2)	2,189 (0.7)
Psoriasis	44 (0.7)	315 (0.5)	163 (0.5)	1,038 (0.3)
Epilepsy	205 (3.3)	768 (1.2)	1,209 (4.0)	4,001 (1.3)
Traumatic brain injury	387 (6.2)	1,443 (2.3)	2,662 (8.7)	8,410 (2.8)
Diabetes	526 (8.4)	4,760 (7.6)	1,706 (5.6)	10,976 (3.6)
Multiple sclerosis	30 (0.5)	184 (0.3)	106 (0.3)	757 (0.2)
Obesity	288 (4.6)	2,130 (3.4)	2,075 (6.8)	12,090 (4.0)

Abbreviations: PTSD, post-traumatic stress disorder; ADHD, attention deficit hyperactivity disorder.

a Most restrictive algorithm.

b Hospitalizations are defined as hospital admissions of more than 12 hours.

Both among individuals who died by suicide and among those who attempted suicide, hospital admission rates at psychiatric and somatic departments were higher than in the reference groups. For example, in the month leading up to the suicide, 7.7% had been admitted to a psychiatric department while 14% had been admitted to a somatic department compared to 0.1% and 1.3% in the reference group (Table 1).

**Figure 1.**
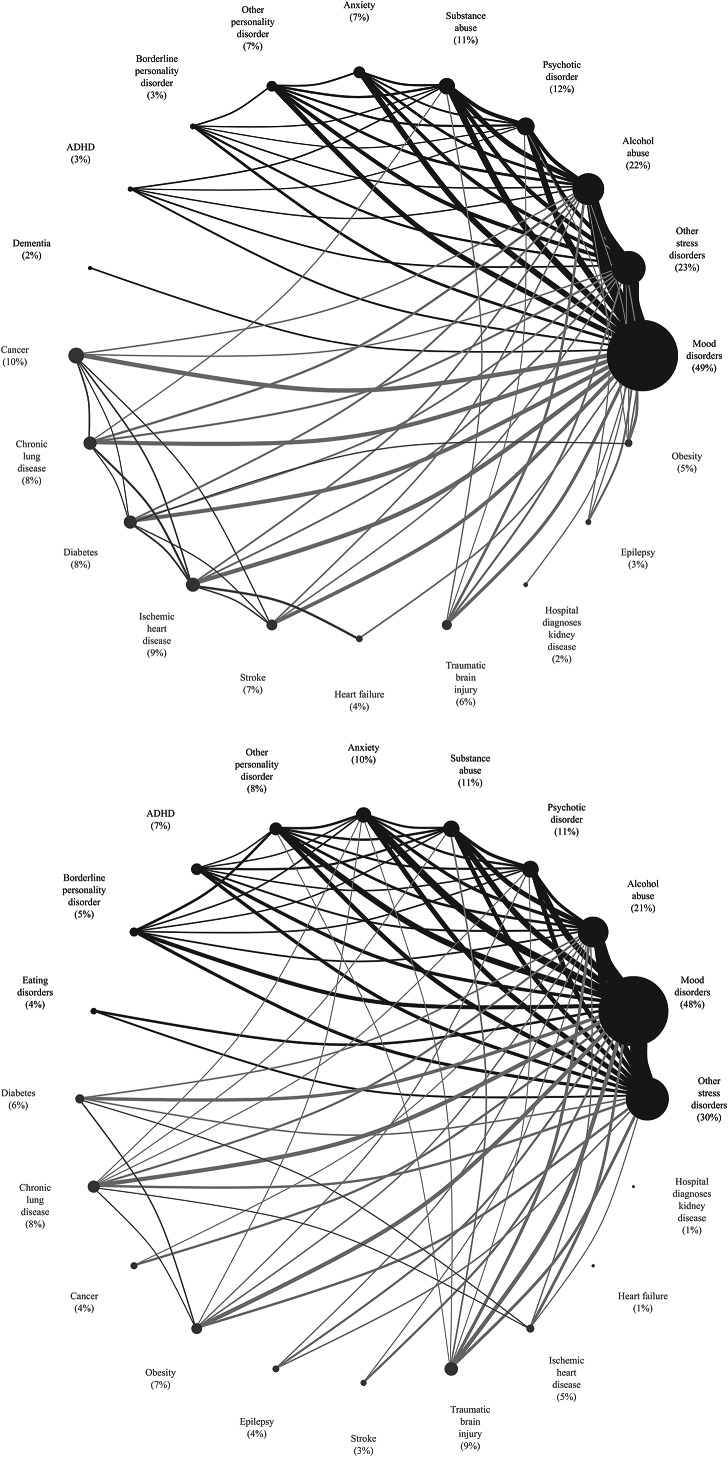
Network graph depicting the 10 most common psychiatric versus somatic diseases (using 10 years of look back) and their internal relationship for (A) individuals who died by suicide and (B) individuals who had a first-ever suicide attempt. Only prespecified psychiatric disorders and somatic diseases were included in this analysis. In the network graph, the proportion of individuals with a given disease is illustrated by the size of the nodes whereas the co-existence of diseases is illustrated by the thickness of the link drawn between the nodes. To avoid over-cluttering of the chord diagram, only diseases co-occurring in more than xx% of individuals were visually linked to each other. Selection of psychiatric disorders and somatic diseases was based on a data-driven exploration of the data set performed for each definition of outcomes, separately. As such, included diseases varied across outcomes.

Across outcomes, the prevalence of single psychiatric comorbidities was high and largely similar as illustrated by the size of the nodes in the network graph (Figure 1). Mood disorders were most common (recorded in approximately half of individuals in both case groups) followed by other stress disorders than post-traumatic stress disorder (23% of those who died by suicide vs. 30% of those who had their first suicide attempt), and alcohol abuse (22% of those who died by suicide vs. 21% of those who had their first suicide attempt). In terms of single somatic diseases, minor differences were observed across outcomes. To this end, cancer and ischemic heart disease were the most prevalent single somatic diseases in those who died by suicide (10% vs. 9%), while traumatic brain injury and chronic lung disease occurred most frequently among those who attempted suicide (9% vs. 8%; Figure 1). Irrespective of outcome, co-existence of psychiatric diseases was common as illustrated by the thickness of the links between nodes in the network graph. More than half (55% vs. 56%) had two or more co-existing psychiatric and/or somatic diseases. Most frequently, mood disorders and other stress disorders co-existed in 18% of individuals who died by suicide and in 19% of those who had their first suicide attempt. Among the most frequent combinations of psychiatric disorders and somatic diseases were mood disorders and cancer (4.8% of those who died by suicide), and mood disorders and traumatic brain injury (4.5% of those who attempted suicide; Figure 1). For both outcomes, the prevalence of psychiatric disorders and somatic diseases, alone or in combination, was several folds higher than in the reference groups (Supplementary Figure S2). Sex-stratified analyses revealed important differences, particularly among those who died by suicide. To this end, the prevalences of the most frequent psychiatric disorders were higher among women, for example, 66% of women who died by suicide had a diagnosis of mood disorders compared to 43% of men (Supplementary Table S3).

**Figure 2.**
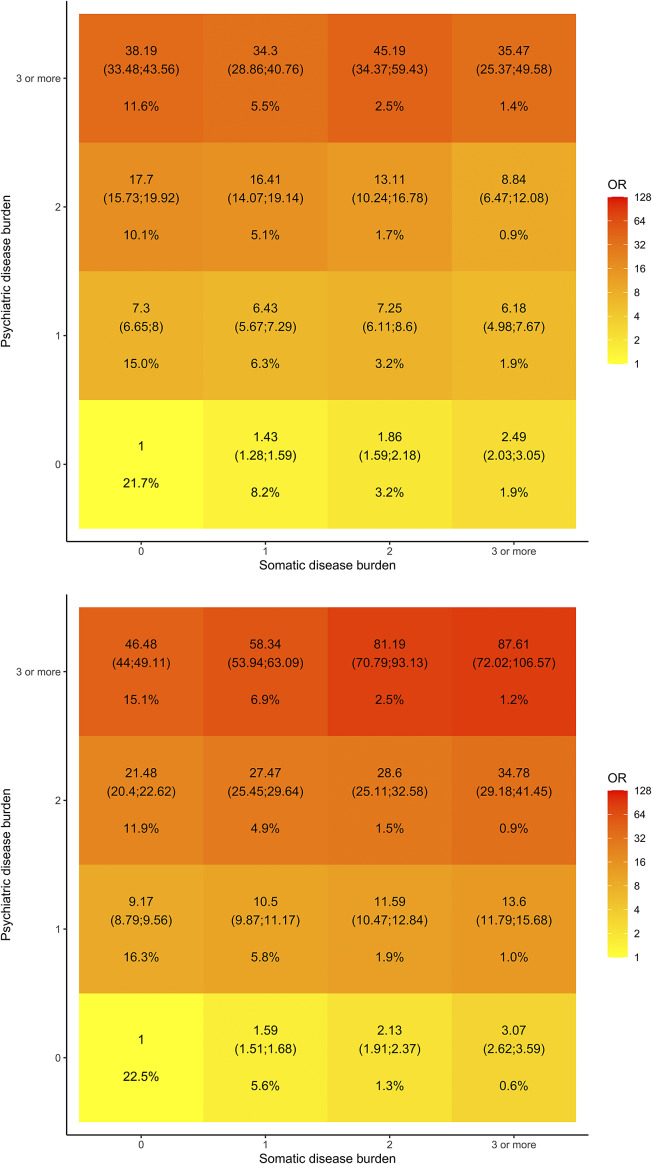
Heat map illustrating the probability of suicidal behavior depending on the burden of psychiatric disorders and somatic diseases in combination, that is, the crude relative odds of experiencing the outcome if having a specific combination of counts of somatic diseases versus psychiatric disorders using as reference the crude relative odds of experiencing the outcome if having 0 somatic diseases and 0 psychiatric disorders. (A) Individuals who died by suicide and (B) individuals who had a first-ever suicide attempt. The number of psychiatric and somatic comorbidities is the total count of prespecified psychiatric versus somatic comorbidities listed under “psychiatric comorbidities” and “somatic comorbidities” in Supplementary Table S1. In each cell of the grid, crude odds ratios (OR) and prevalence proportion are reported. The cells are colored according to log (OR). OR is calculated using conditional logistic regression.

When investigating the association between the burden of comorbidities, we found generally increasing relative odds of attempting suicide the higher the combined burden of somatic and psychiatric disease. To this end, the odds of attempting suicide was 88-fold higher among those who had three or more psychiatric and somatic comorbidities relative to those who had zero comorbidities (Figure 2). Similarly, the odds of dying by suicide was 35-fold higher among those with three or more psychiatric and somatic comorbidities. The burden of somatic diseases did, however, not seem to augment the association as observed among those who attempted suicide (Figure 2). The observed increase in relative odds of suicide and suicide attempt was evident for both sexes, though most pronounced for women who died by suicide (Supplementary Figure S3). This potential modifying effect of somatic diseases on the risk of suicide was further investigated in a *post hoc* analysis. We found a slightly lower crude OR in individuals with at least one somatic disease, for example, if having two psychiatric diseases, the crude OR was 14 if having at least one somatic disease while the crude OR was 18 in the absence of somatic diseases. Stratifying by age groups, the presence of somatic diseases seemed to have a protective effect in individuals older than 40 years (e.g., crude OR 18 if zero somatic diseases and 2 psychiatric diseases vs. crude OR 12 in the presence of somatic disease), whereas the opposite pattern emerged for young adults (Table 2).

**Table 2.** Tabulation of the probability of suicide according to the number of psychiatric disorders and presence of somatic diseases in the overall population and stratified by age groups

Age group	Somatic diseases	Number of psychiatric disorders			
		0 OR (95% CI)	1 OR (95% CI)	2 OR (95% CI)	3+ OR (95% CI)
All	No	1.00 (ref)	7.29 (6.65;8.00)	17.70 (15.73;19.92)	38.25 (33.54;43.63)
	Yes	1.61 (1.47;1.77)	6.58 (5.93;7.29)	14.15 (12.45;16.07)	36.86 (32.02;42.42)
10–39 years (*N* = 12,815)	No	1.00 (ref)	5.58 (4.53;6.88)	11.97 (9.35;15.33)	34.07 (27.13;42.79)
	Yes	1.26 (0.93;1.71)	7.40 (5.30;10.35)	14.58 (10.11;21.03)	42.88 (31.67;58.08)
40–54 years (*N* = 19,935)	No	1.00 (ref)	8.97 (7.59;10.59)	23.73 (19.29;29.18)	48.05 (38.66;59.71)
	Yes	1.23 (0.99;1.54)	8.85 (7.11;11.01)	20.28 (15.62;26.33)	46.22 (36.06;59.25)
≥55 years (*N* = 35,253)	No	1.00 (ref)	7.07 (6.19;8.08)	17.54 (14.63;21.02)	31.96 (24.57;41.56)
	Yes	1.70 (1.51;1.90)	5.97 (5.25;6.79)	12.44 (10.57;14.64)	28.41 (23.01;35.07)

*Note*: The odds ratios (ORs) are calculated using logistic regression using the combination of 0 psychiatric disorders and absence of somatic diseases as reference group, including somatic diseases, psychiatric disorders, and the combination hereof as an interaction term.

Acknowledging some level of misclassification of suicide attempts in the Danish health care registries, we applied a less restrictive definition of suicide attempts in a sensitivity analysis. This revealed more conservative findings, for example, a lower proportion of women, less prevalent psychiatric disorders, and lower crude ORs of the combined burden of psychiatric and somatic comorbidities, though the general tendencies remained unchanged (data not shown).

## Discussion

In this observational study, we mapped co-existence of the most prevalent psychiatric and somatic diseases and estimated the influence of the increasing burden of psychiatric and somatic comorbidities on the risk of having suicidal behavior. We found that more than half of individuals with suicidal behavior had at least two co-existing psychiatric and/or somatic diseases, most commonly psychiatric disorders like mood disorders and stress disorders, and somatic disorders like cancer and traumatic brain injury. We also found higher relative odds of suicidal behavior the higher the combined burden of somatic and psychiatric comorbidities. *Post hoc* analysis revealed, however, a potential protective effect of the presence of somatic diseases on the risk of dying by suicide in the older age groups.

### Strengths and limitations

The Danish healthcare registries cover data on hospital admissions and drug use on all Danish citizens irrespective of age, sex, and income. This provides unique opportunities to perform observational, population-based suicide research on high-quality data covering an entire nation. Of note, the registries are built for administrative purposes, and limitations should be considered, mainly related to misclassification of outcomes and variables. Misclassification of suicide is minimal in the Danish Cause of Death registry [13]. For suicide attempts, some misclassification is known to occur. The most restrictive definition used in the main analysis has a positive predictive value of 73% [10], and is widely accepted in Danish registry-based suicide research. Differential misclassification may occur, that is, that suicidal behavior is more likely to be registered as such in individuals with existing psychiatric diseases. This would overestimate the crude ORs illustrated in the heat map, though unlikely to an extent that explains the magnitude of the crude ORs observed in this study. Similarly, it cannot be ruled out that suicidal behavior is more likely to be registered as such if the number of somatic diseases increases. If such misclassification is present, our estimates are conservative of the potential protective effect of somatic diseases on suicide risk among the oldest.

The completeness of recordings in the health registries is generally good, and the recording of deaths in the Cause of Death registry is complete. [9]. Some under-recording of suicide attempts is known to occur. Individuals who do not seek medical attention following a suicide attempt are not covered or might be incorrectly registered as having had an accident or another illness. Some reluctance towards properly registering suicidal attempt as the cause of contact exists [14], that is, suicidal attempts might be recorded as a drug overdose or accident. To meet this, we did a sensitivity analysis using less restrictive definitions including certain codes indicating drug overdose or accidents. This led to more conservative crude estimates of OR related to the combined burden of comorbidities likely indicating some washout of the results due to a higher level of misclassification. While the recording of chronic diseases like diabetes or epilepsy that requires specific medical treatment or intervention in the hospital is close to complete, other less abruptly occurring chronic diseases like obesity or mild depression may be under-recorded. As such, this study presents a conservative estimate of the co-existence of diseases, which in reality is probably aggravated even further in psychiatric patients in whom somatic diseases are underdiagnosed [15].

### Comparison with existing literature

The combined burden of co-existing mental disorders and physical illnesses in individuals with suicidal behavior is sparsely elucidated in existing literature. Indirectly supporting the validity of our results is the previously observed high prevalences of mental disorders and physical illnesses when evaluated separately. In accordance with our findings, a meta-analysis suggests that mental disorders are present in up to 71% of 35–65-year-olds who die by suicide [16], while a case–control study from the United States found that physical illnesses were present in 62% of those who died by suicide. In addition, they found that physical multimorbidity (two or more conditions) was associated with a 4-fold increase in the risk of suicide [17]. As isolated risk factors, physical illnesses like cancer, stroke, and chronic obstructive pulmonary disease also seem to increase the risk of suicidal behavior [17–20] though less than mental disorders like major depressive disorders and bipolar diseases, where the risk of suicide is increased up to 9-fold [21].

In the present study, an 88-fold increase in the odds of suicide attempt and a 35-fold increase in the odds of suicide was observed among individuals who had three or more of both psychiatric and somatic comorbidities relative to those who had none. This has not been shown before but is indirectly supported by a study indicating that suicide risk was particularly elevated if somatic diseases and psychiatric disorders were diagnosed close in time to each other [22]. Altogether, this indicates that the combined burden of disease is an important measure in the prediction of suicidal behavior. It is, however, puzzling that the presence of somatic diseases seems to have a potentially protective effect on the risk of suicide in the oldest age groups, though the same cannot be found for suicide attempts.

### Clinical implications and future perspectives

The high prevalences of psychiatric and somatic comorbidities observed in this study underpin that these are important components in suicide risk algorithms, particularly if being present at the same time. The preventive potential hereof should be investigated, for example through calculations of absolute risk increases and attributable proportions related to the most frequent combinations of psychiatric and somatic diseases, for example, mood disorders and cancer in the oldest, and mood disorders and traumatic brain injury in the younger age groups. From a clinical perspective, our results emphasize the need for enhanced awareness of suicidal risk across both somatic and psychiatric departments and suggest that close collaborations between psychiatric and somatic departments as well as across primary and secondary care are warranted to prevent suicidal behavior.

The ambiguous finding that somatic diseases potentially protect against suicide in the oldest multimorbid patient is of interest. This might indicate a collateral benefit of the more intensive health care and treatment demanded by those with a higher burden of somatic diseases. An alternative explanation is that older individuals with a high burden of disease and functional impairment are taken care of at nursing home facilities. Since nursing homes permit 24-hour care and frequent interventions between residents and staff, low suicide rates could be expected. Only few studies have investigated this and with ambiguous results [23, 24]. Finally, a depletion of individuals susceptible to suicide may explain that the increased risk related to disease burden vanished with age. Irrespective of the underlying reason, however, it is puzzling that the same pattern does not seem to emerge in individuals with suicide attempt.

## Conclusion

Co-existing psychiatric and somatic comorbidities are common in individuals with suicidal behavior. Higher combined burden of psychiatric and somatic comorbidities increased the odds of suicidal behavior, though the presence of somatic diseases had a potential protective effect on the risk of suicide in older individuals. These findings support that both co-existence and burden of somatic and psychiatric comorbidities are important components in suicide risk assessment and warrant collaboration and enhanced awareness of suicidal behavior risks across somatic and psychiatric departments.

## Supporting information

Reilev et al. supplementary material Reilev et al. supplementary material

## Data Availability

According to Danish law, individual-level data cannot be shared by the authors.

## Supplementary material

The supplementary material for this article can be found at http://doi.org/10.1192/j.eurpsy.2024.1781.
