# Supplementary material for: Burden of psychiatric and somatic comorbidities in individuals with suicidal behavior: a nationwide Danish registry-based, observational study
Source: Eur Psychiatry. 2025 Jan 21;68(1):e23. doi: 10.1192/j.eurpsy.2024.1781 (PMC11822957; doi:10.1192/j.eurpsy.2024.1781)
Supplement: Reilev et al. supplementary material [file S0924933824017814sup001.docx]

# Supplementary material.

**e-Table 1.** ICD-10 codes used to define suicide attempts and suicide.

| **Outcomes** | **ICD-10 codes** | **NOMESCO code** |
| --- | --- | --- |
| Suicide | X60 X61 X62 X63 X64 X65 X66 X67 X68 X69 X70 X71 X72 X73 X74 X75 X76 X77 X78 X79 X80 X81 X82 X83 X84 |  |
| Suicide attempt, specific | X60 X61 X62 X63 X64 X65 X66 X67 X68 X69 X70 X71 X72 X73 X74 X75 X76 X77 X78 X79 X80 X81 X82 X83 X84 | 4 |
| Suicide attempt, sensitive | X60 X61 X62 X63 X64 X65 X66 X67 X68 X69 X70 X71 X72 X73 X74 X75 X76 X77 X78 X79 X80 X81 X82 X83 X84  And a main diagnosis of F00-F99 in combination with at least one of the following T42 T43 T58 T39 T40 (excl. T401) T71 | 4 |

**e-Table 2.** List of prespecified psychiatric disorders and somatic disease entities, and the corresponding ICD-10- and ATC-codes used to define the diseases from the registries. Whenever relevant the disease entity is defined by either diagnosis codes recorded from the in- and outpatient setting or the filling of a prescription at the community pharmacy.

|  | **ICD-10 codes** | **ATC codes** |
| --- | --- | --- |
| ***Psychiatric disease entities*** |  |  |
| Mood disorders* | F3 | N06A |
| Anxiety | F40 F41 |  |
| PTSD | F431 |  |
| Other stress disorders | F43 (excl. F431) |  |
| Obsessive compulsive disorder | F42 |  |
| Psychotic disorder | F2 |  |
| ADHD | F90 |  |
| Eating disorder | F50 |  |
| Substance abuse* | F11 F12 F13 F14 F15 F16 F18 F19 | N07BC |
| Alcohol abuse* | E244 E529A F10 G312A G312B G312C G312D G312E G405B G621 G721 I426 K292 K70 K852 K860 O354 P043 T519 Z502 Z714 Z721 | N07BB |
| Dementia* | B220A F00 F01 F02 F03 F1073 F1173 F1273 F1373 F1473 F1573 F1673 F1873 F1973 G30 G31 | N06D |
| Borderline personality disorder | F603 |  |
| Other personality disorder | F60 (excl. F603) |  |
| Autism spectrum disorders | F84 |  |
| ***Somatic disease entities*** |  |  |
| Chronic lung disease* | J41 J42 J43 J44 J45 J46 J47 E84 | R03AC12 R03AC13 R03AC18 R03AC19 R03AK R03AL R03BA R03BB04 R03BB05 R03BB06 R03BB07 R03CC12 |
| Ischemic heart disease* | I20 I21 I22 I23 I24 I25 | B01AC24 C01DA N02BA |
| Heart failure | I099A I110 I130 I132 I50 |  |
| Stroke | I60 I61 I62 I63 I64 I69 |  |
| Cancer | C (excl. C44 C98 C99) |  |
| Inflammatory bowel disease | K50 K51 M074 M075 M091 |  |
| Rheumatoid arthritis | M05 M06 |  |
| Hospital-diagnoses kidney disease | E102 E112 E142 I12 I13 N00 N01 N02 N03 N04 N05 N07 N08 N11 N14 N18 N19 Z992 Z49 (or procedure codes BJFZ BJFD) |  |
| Psoriasis | L40 |  |
| Epilepsy | G40 |  |
| Traumatic brain injury | S06 |  |
| Diabetes* | E10 E11 E13 E14 G632 H360 N083 O24 (excl. O244) | A10 |
| Multiple sclerosis | G35 |  |
| Obesity* | E66 | A08 |

* Covariate defining algorithm identifies individuals recorded with mentioned discharge diagnosis(es) OR the redeemed drug(s)

**e-Table 3**. Baseline characteristics, stratified by sex.

|  | **Suicide**  **men** | **Suicide**  **women** | **Suicide attempt men*** | **Suicide attempt women*** |
| --- | --- | --- | --- | --- |
|  | 4,559 | 1,698 | 12,208 | 18,362 |
| **Demographics** | |  |  |  |
| Age [IQR] | 55 [43-68] | 57 [44-70] | 39 [24-53] | 24 [17-47] |
| 70+ years | 1,052 (23.1) | 428 (25.2) | 1,046 (8.6) | 1,189 (6.5) |
| 55-69 years | 1,236 (27.1) | 509 (30.0) | 1,780 (14.6) | 1,770 (9.6) |
| 40-54 years | 1,408 (30.9) | 446 (26.3) | 3,118 (25.5) | 3,205 (17.5) |
| 23-39 years | 667 (14.6) | 230 (13.5) | 3,533 (28.9) | 3,487 (19.0) |
| 10-22 years | 196 (4.3) | 85 (5.0) | 2,731 (22.4) | 8,711 (47.4) |
| **Socioeconomics** | |  |  |  |
| Income |  |  |  |  |
| <1. quartile | 1,461 (32.0) | 467 (27.5) | 4,278 (35.1) | 5,364 (29.2) |
| 1.-2. quartile | 1,180 (25.9) | 404 (23.8) | 3,235 (26.5) | 3,845 (20.9) |
| 2.-3. quartile | 807 (17.7) | 337 (19.8) | 2,041 (16.7) | 3,359 (18.3) |
| 3.-4. quartile | 601 (13.2) | 244 (14.4) | 1,419 (11.6) | 3,178 (17.3) |
| >4. quartile | 510 (11.2) | 246 (14.5) | 1,232 (10.1) | 2,615 (14.2) |
| Married | 1,485 (32.6) | 504 (29.7) | 2,740 (22.4) | 3,038 (16.5) |
| Unmarried | 3,074 (67.4) | 1,194 (70.3) | 9,468 (77.6) | 15,324 (83.5) |
| Educational level |  |  |  |  |
| Primary school | 1,663 (36.5) | 623 (36.7) | 6,466 (53.0) | 11,615 (63.3) |
| High school or vocational | 1,961 (43.0) | 575 (33.9) | 4,099 (33.6) | 4,357 (23.7) |
| Further education or bachelor | 548 (12.0) | 355 (20.9) | 831 (6.8) | 1,513 (8.2) |
| Master or PhD | 264 (5.8) | 89 (5.2) | 298 (2.4) | 253 (1.4) |
| Unknown | 122 (2.7) | 56 (3.3) | 508 (4.2) | 612 (3.3) |
| Occupation |  |  |  |  |
| Student | 190 (4.2) | 74 (4.4) | 1,976 (16.2) | 7,497 (40.8) |
| Retired | 2,003 (43.9) | 952 (56.1) | 3,207 (26.3) | 3,631 (19.8) |
| Working | 1,627 (35.7) | 393 (23.1) | 3,964 (32.5) | 3,613 (19.7) |
| Unknown | 739 (16.2) | 279 (16.4) | 3,060 (25.1) | 3,621 (19.7) |
| **History of psychiatric disease** | | |  |  |
| Hospitalization at psychiatric department during previous year** | 899 (19.7) | 478 (28.2) | 2,622 (21.5) | 3,566 (19.4) |
| Hospitalization at psychiatric department within previous 30 days** | 299 (6.6) | 185 (10.9) | 1,055 (8.6) | 1,299 (7.1) |
| Mood disorders | 1,970 (43.2) | 1,125 (66.3) | 5,473 (44.8) | 9,324 (50.8) |
| Anxiety | 261 (5.7) | 208 (12.2) | 976 (8.0) | 2,046 (11.1) |
| PTSD | 61 (1.3) | 44 (2.6) | 286 (2.3) | 434 (2.4) |
| Other stressdisorders than PTSD | 932 (20.4) | 521 (30.7) | 3,178 (26.0) | 5,843 (31.8) |
| Obsessive compulsive disorder | 32 (0.7) | 25 (1.5) | 167 (1.4) | 454 (2.5) |
| Psychotic disorders | 504 (11.1) | 232 (13.7) | 1,514 (12.4) | 1,828 (10.0) |
| ADHD | 130 (2.9) | 44 (2.6) | 1,067 (8.7) | 1,148 (6.3) |
| Eating disorder | 6 (0.1) | 60 (3.5) | 41 (0.3) | 1,068 (5.8) |
| Substance abuse | 461 (10.1) | 213 (12.5) | 1,999 (16.4) | 1,288 (7.0) |
| Alcohol abuse | 1,016 (22.3) | 335 (19.7) | 3,542 (29.0) | 2,814 (15.3) |
| Dementia | 94 (2.1) | 36 (2.1) | 167 (1.4) | 142 (0.8) |
| Borderline personality disorder | 53 (1.2) | 151 (8.9) | 243 (2.0) | 1,412 (7.7) |
| Other personality disorders than borderline | 247 (5.4) | 169 (10.0) | 794 (6.5) | 1,693 (9.2) |
| Autism | 51 (1.1) | 25 (1.5) | 398 (3.3) | 654 (3.6) |
| **History of somatic diseases** | | |  |  |
| Hospitalization at somatic department during previous year** | 1,453 (31.9) | 697 (41.0) | 4,083 (33.4) | 6,300 (34.3) |
| Hospitalization at somatic department within previous 30 days** | 589 (12.9) | 300 (17.7) | 1,924 (15.8) | 3,052 (16.6) |
| Chronic lung disease | 337 (7.4) | 192 (11.3) | 877 (7.2) | 1,467 (8.0) |
| Ischemic heart disease | 419 (9.2) | 143 (8.4) | 840 (6.9) | 622 (3.4) |
| Heart failure | 193 (4.2) | 47 (2.8) | 300 (2.5) | 145 (0.8) |
| Stroke | 297 (6.5) | 129 (7.6) | 577 (4.7) | 476 (2.6) |
| Cancer | 451 (9.9) | 193 (11.4) | 600 (4.9) | 653 (3.6) |
| Inflammatory bowel disease | 79 (1.7) | 23 (1.4) | 155 (1.3) | 243 (1.3) |
| Rheumathoid arthritis | 33 (0.7) | 29 (1.7) | 60 (0.5) | 150 (0.8) |
| Hospital diagnosed kidney disease | 112 (2.5) | 41 (2.4) | 204 (1.7) | 168 (0.9) |
| Psoriasis | 30 (0.7) | 14 (0.8) | 71 (0.6) | 92 (0.5) |
| Epilepsy | 122 (2.7) | 83 (4.9) | 523 (4.3) | 686 (3.7) |
| Traumatic brain injury | 286 (6.3) | 101 (5.9) | 1,318 (10.8) | 1,344 (7.3) |
| Diabetes | 402 (8.8) | 124 (7.3) | 867 (7.1) | 839 (4.6) |
| Multiple sclerosis | 16 (0.4) | 14 (0.8) | 36 (0.3) | 70 (0.4) |
| Obesity | 152 (3.3) | 136 (8.0) | 516 (4.2) | 1,559 (8.5) |

PTSD: Post traumatic stress disorder; ADHD: Attention deficit hyperactivity disorder

**e-figure 1.** Design diagram visualizing study design and inclusion and exclusion criteria

**e-figure 2.** Network graph depicting the 10 most common psychiatric disorders and somatic diseases (using 10-years of look back) and their internal relationship for A) the reference group for individuals who die by suicide, B) the reference group for individuals who have a first-ever suicide attempt. Only prespecified psychiatric disorders and somatic diseases were included in this analysis. In the network graph the proportion of individuals with a given disease is illustrated by the size of the nodes whereas the co-existence of diseases is illustrated by the thickness of the link drawn between the nodes. To avoid over-cluttering of the chord diagram, only diseases co-occurring in more than 1% of individuals were visually linked to each other. Selection of psychiatric disorders and somatic diseases was based on a data driven exploration of the data set performed for each definition of outcomes, separately. As such, included diseases varied across outcomes.

**e-Figure 3.** Heat map illustrating the probability of suicidal behavior depending on the burden of psychiatric disorders and somatic diseases in combination i.e., the crude relative odds of experiencing the outcome if having a specific combination of counts of somatic diseases vs. psychiatric disorders using as reference the crude relative odds of experiencing the outcome if having 0 somatic diseases and 0 psychiatric disorders. A) men who died by suicide, B) women who died by suicide, C) men who had a first-ever suicide attempt, and D) women who had a first-ever suicide attempt.

The number of psychiatric and somatic comorbidities is the total count of prespecified psychiatric vs. somatic comorbidities listed under “psychiatric comorbidities” and “somatic comorbidities” in e-Table 1. In each cell of the grid, crude odds ratios (OR) and prevalence proportion is reported. The cells are colored according to log (OR). OR is calculated using conditional logistic regression.
